# Association between number of parathyroid glands identified during total thyroidectomy and functional parathyroid preservation

**DOI:** 10.1007/s00423-021-02287-6

**Published:** 2021-08-18

**Authors:** Fiona Riordan, Matthew S. Murphy, Linda Feeley, Patrick Sheahan

**Affiliations:** 1 Department of Otolaryngology, Head and Neck Surgery, South Infirmary Victoria University Hospital, Cork, T12 Ireland; 2 Department of Endocrinology, South Infirmary Victoria University Hospital, Cork, Ireland; 3 Department of Pathology, Cork University Hospital, Cork, Ireland; 4 ENTO Research Unit, University College Cork, Cork, Ireland

**Keywords:** Thyroidectomy, Parathyroid identification, Parathyroid, Hypocalcemia, Hypoparathyroidism

## Abstract

**Purpose:**

Systematic identification of all 4 parathyroid glands has been recommended during total thyroidectomy (TT); however, it is unclear whether this strategy necessarily translates into optimized functional parathyroid preservation. We wished to investigate the association between number of parathyroids identified intraoperatively during TT, and incidence of incidental parathyroidectomy, and postoperative hypoparathyroidism.

**Methods:**

Retrospective review of prospectively maintained database of 511 consecutive patients undergoing TT at an academic teaching hospital. The association between number of parathyroid glands identified intraoperatively and incidence of biochemical hypocalcaemia (defined as any calcium < 2 mmol/L *n* first 48 h after surgery), symptomatic hypocalcaemia; permanent hypoparathyroidism (defined as any hypocalcaemia or need for calcium or vitamin D > 6 months after surgery), and incidental parathyroidectomy, was investigated. The association between number of parathyroid glands visualized and postoperative parathyroid hormone (PTH) levels was investigated in a subset of 454 patients.

**Results:**

Patients in whom a greater number of parathyroids had been identified had a significantly higher incidence of biochemical and symptomatic hypocalcaemia, and significantly lower postoperative PTH levels, than patients with fewer glands identified. There were no significant differences in incidence of permanent hypoparathyroidism or incidental parathyroidectomy. On multivariate analysis, malignancy, Graves disease, and identification of 3–4 parathyroids were independent predictors of biochemical hypocalcaemia. For symptomatic hypocalcaemia, identification of 2–4 parathyroids, and identification of 3–4 parathyroids, were significant.

**Conclusions:**

Systematic identification of as many parathyroid glands as possible during TT is not necessary for functional parathyroid preservation.

**Supplementary Information:**

The online version contains supplementary material available at 10.1007/s00423-021-02287-6.

## Introduction

Hypoparathyroidism after total thyroidectomy (TT) may arise due to inadvertent removal of parathyroid tissue during surgery, or due to intraoperative trauma or damage to vasculature supplying parathyroid glands. The reported incidence of hypoparathyroidism after thyroidectomy varies widely, according to cohort studied, surgical technique, and definition used for hypoparathyroidism [1]. Temporary hypoparathyroidism, which self-resolves within 6 months without need for calcium or vitamin D supplementation, is reported to occur after 20–70% of TTs. Permanent hypoparathyroidism indicates hypocalcaemia beyond 6 months, and/or need for calcium or vitamin D supplementation to maintain normocalcaemia, and is reported after 1–9% of cases [2–7], but with rates up to 18% reported [8, 9].

Previously reported risk factors for post-thyroidectomy hypoparathyroidism include surgery for malignancy [6, 9], central neck dissection (CND) [3, 4, 6, 9], and incidental parathyroidectomy [3, 9–11]. In addition, many previous authors have reported routine identification of all 4 parathyroid glands to be important to minimize the risk of hypoparathyroidism [5, 9, 12–15]. However, it is possible that the extra dissection required to identify some parathyroid glands may lead to compromise of their blood supply, particularly if such dissection leads to bleeding which needs to be controlled by electrocautery or other means. Other authors have not found identification of a higher number of parathyroid glands to be associated with reduced risk of postoperative hypoparathyroidism [3, 10, 16, 17]. Therefore it is not clear whether systematic identification of as many parathyroid glands as possible necessarily translates into optimized functional parathyroid preservation.

The purpose of the present paper was to investigate the impact of intraoperative parathyroid identification on risk of incidental parathyroidectomy, early postoperative hypocalcaemia and permanent hypoparathyroidism in a large cohort of patients undergoing TT.

## Methods

Approval to perform the study was obtained from the Cork Clinical Research Ethics Committee. The study consisted of a retrospective review of a prospectively maintained database of patients undergoing thyroidectomy between April 2009 and February 2020. Inclusion criteria for the study were all patients undergoing TT during the study period. Exclusion criteria were concomitant parathyroidectomy, preoperative hypercalcaemia, or concomitant central neck dissection.

Clinical information was extracted from the prospectively maintained database, retrospective review of patient charts, and consultation of laboratory information system. Information extracted from the database included surgical details, number of parathyroids identified intraoperatively, and postoperative occurrence of symptoms of hypocalcaemia.

Thyroidectomy was performed using capsular dissection technique [17]. Briefly, after retraction of strap muscles and elevation of thyroid superior and inferior poles, soft tissue is meticulously reflected off the thyroid capsule to be preserved in situ. Close attention is paid to detect any parathyroids located on or underneath the thyroid capsule, or located close to the thyroid in the vicinity of the tubercle of Zuckerkandl and recurrent laryngeal nerve. Where these are detected, great care is taken to reflect them away from thyroid, as much as possible preserving feeding blood vessels intact. Where parathyroids are not apparent, they are not systematically sought, but rather assumed to be within the soft tissue being reflected off the thyroid capsule, or within fatty tissue lateral or deep to the thyroid.

Parathyroids were considered to be positively identified based on characteristic color (brown/caramel), consistency (firm, non-friable), and shape (round or flattened). We only considered parathyroids to be positively identified when the above criteria were met unequivocally; “presumed” parathyroids were not counted as identified. At the end of the operation, a note was made by the surgeon of the number of glands which were definitely identified and preserved, as well as any glands removed, reimplanted, or left in situ but considered to have been devascularized. These findings were recorded in the thyroid database.

Postoperative management of patients during the period of this study involved a 2-night hospital stay, with calcium levels taken at 6 a.m. and 3 p.m. on postoperative day 1, and at 6 a.m. on postoperative day 2. Patients were not routinely prescribed calcium or vitamin D perioperatively. Oral or intravenous calcium was generally only administered to patients with symptomatic hypocalcaemia, or significant biochemical hypocalcaemia lasting several days.

The total number of parathyroids identified in each case was extracted from the thyroid database. The number of parathyroids preserved in situ was defined as the total number of parathyroids identified in each case, minus removed, devascularized or reimplanted glands. Biochemical hypocalcaemia was defined as any uncorrected calcium level < 2.0 mmol/L on either day 1 or day 2. Symptomatic hypocalcaemia was defined as presence of any paraesthesia, numbness, or muscle cramps, during the hospital stay, and was recorded in the prospective database. Postoperatively, patients with hypocalcaemia were followed until they were off calcium and vitamin D and calcium levels had returned to normal. Permanent hypoparathyroidism was defined as any calcium level < 2.0 mmol/L or need to take either calcium or vitamin D more than 6 months after the surgery.

During histological examination, the posterior surface of TT specimens is carefully examined to identify any possible parathyroid glands (yellow/brown ovoid structures, 2 to 3 mm in size), which are submitted for histological examination. Incidental parathyroidectomy is confirmed in all cases based on the microscopic identification of parathyroid tissue.

Statistical analysis was performed using XLSTAT (Addinsoft, Paris, France). The impact of number of parathyroid glands identified intraoperatively on outcomes was analyzed in binary fashion by combining cases with more or less parathyroids than the index number of glands identified being studied, and this process repeated for each index cutoff. That is, outcomes were compared for cases with 0 versus 1–4 glands identified, 0–1 versus 2–4 glands identified, and so on. A Fisher exact test was performed on 2 × 2 contingency tables. For comparison of normally distributed data, a Student’s *t*-test was used. Impact of factors on hypocalcaemia outcomes was studied using odds ratios, and multivariate analysis performed using backwards logistic regression.

## Results

During the study period, 592 patients underwent TT. Eighty-one were excluded due to undergoing concomitant central neck dissection (70) excision of parathyroid adenoma (4) or having preoperative hypercalcaemia (7), leaving a final study population of 511 patients. Baseline patient demographics and clinical information is given in Table 1.

**Table 1:** Baseline characteristics of patients

Sex	Male	64
	Female	447
Age	< 18 years old	4
	18–65 years old	401
	> 65 years old	106
Indication for surgery	Graves disease	132
	Non-Graves hyperthyroidism	34
	Malignancy	101
	Suspicious nodule	61
	Multinodular goiter or dominant nodule	183
Cancer diagnosis	Papillary	90*
	Follicular/Hurthle cell	11
	Medullary	1
	Anaplastic/poorly differentiated	5
Specimen weight	< 100 g	374
	≥ 100 g	101
	Not known	36

^*^Includes 6 patients with incidental carcinoma in Graves disease

In 64 patients (12.5%), no definitive parathyroid was identified. One, 2, 3, and 4 parathyroids were definitively identified in 97 (19.0%), 124 (24.3%), 118 (23.1%), and 108 (21.1%) of patients respectively. Forty patients underwent intraoperative parathyroid autotransplantation. In 16 cases, parathyroids left in situ were considered to be compromised, and 2 patients had parathyroids removed which were not reimplanted. Considering parathyroids identified and preserved in situ, the number of patients with 0, 1, 2, 3, and 4 glands identified was 75 (14.7%), 99 (19.4%), 129 (25.2%), 121 (23.7%), and 87 (17.0%), respectively.

The overall incidence of biochemical hypocalcaemia, symptomatic hypocalcaemia, and permanent hypoparathyroidism was 30.7%, 14.7%, and 3.1%, respectively. In 71 (13.9%) patients, parathyroid was detected histologically in the surgical specimen.

Table 2 gives the incidence of hypocalcaemia and incidental parathyroidectomy according to total number of parathyroids identified. *p*-values for Fisher exact test between contiguous groupings are given in Table 3. There was a significantly lower incidence of biochemical hypocalcaemia among patients with 0–1 versus 2–4 parathyroids identified (*p* = 0.02), with 0–2 versus 3–4 parathyroids identified (*p* = 0.003), and between patients with 0–3 versus 4 parathyroids identified (*p* = 0.01). The difference between patients with no parathyroid identified versus 1–4 parathyroids identified was just outside significance (*p* = 0.06). For symptomatic hypocalcaemia, there was a significantly lower incidence among patients with 0 versus 1–4 parathyroids identified (*p* = 0.004), between patients with 0–1 versus 2–4 parathyroids identified (*p* = 0.0001), and between patients with 0–2 versus 3–4 parathyroids identified (*p* = 0.01). The difference between patients with 0–3 versus 4 parathyroids identified (*p* = 0.22) was not significant. There were no differences in incidence of permanent hypoparathyroidism or incidental parathyroidectomy according to the number of parathyroids identified.

**Table 2:** Incidence of biochemical and symptomatic hypocalcaemia and permanent hypoparathyroidism according to total number of parathyroid glands identified, and according to number of parathyroid glands identified and preserved in situ

Total number of parathyroid glands identified							
Number of parathyroids	*N*	Biochemical hypoCa	Symptomatic hypoCa	Permanent hypoPTH	Parathyroid in specimen	Preop PTH (ng/L)	Postop PTH (ng/L)
0	64	13 (20.3%)	2 (3.2%)	1 (1.6%)	9 (14.1%)	52.0 ± 30.1	30.0 ± 16.1
1	97	25 (25.8%)	8 (8.2%)	5 (5.2%)	16 (16.5%)	49.8 ± 24.4	29.4 ± 23.2
2	124	34 (27.4%)	21 (16.9%)	3 (2.4%)	16 (12.9%)	51.7 ± 23.0	27.2 ± 19.7
3	118	41 (34.7%)	24 (20.3%)	3 (2.5%)	16 (13.6%)	46.7 ± 24.2	22.3 ± 14.5
4	108	44 (40.7%)	20 (18.5%)	4 (3.7%)	14 (13.0%)	50.9 ± 27.5	20.6 ± 12.6
Number of parathyroid glands identified and preserved in situ							
Number of parathyroids	*N*	Biochemical hypoCa	Symptomatic hypoCa	Permanent hypoPTH	Parathyroid in specimen	Preop PTH (ng/L)	Preop PTH (ng/L)
0	75	17 (22.7%)	4 (5.3%)	3 (2.7%)	10 (13.3%)	50.4 ± 29.1	28.1 ± 16.5
1	99	27 (27.7%)	10 (10.1%)	4 (4.0%)	18 (18.1%)	49.6 ± 24.8	29.7 ± 23.2
2	129	36 (27.9%)	24 (18.6%)	3 (2.3%)	17 (13.2%)	53.8 ± 24.8	26.3 ± 20.0
3	121	44 (36.3%)	24 (19.8%)	4 (3.3%)	16 (13.2%)	43.7 ± 20.3	21.4 ± 13.3
4	87	33 (37.9%)	13 (14.9%)	3 (3.4%)	10 (11.5%)	52.6 ± 28.5	22.6 ± 12.7

*HypoCa*, hypocalcaemia;

*HypoPTH*, hypoparathyroidism;

*PTH*, parathyroid hormone

**Table 3:** *p*-values (Fisher exact test) for comparisons of hypocalcaemia outcomes between groupings, according to total number of parathyroid glands identified, and according to number of parathyroid glands identified and preserved in situ

Number of parathyroids	Biochemical hypoCa	Symptomatic hypoCa	Permanent hypoPTH	Parathyroid in specimen	Preop PTH	Postop PTH
Total number of parathyroid glands identified						
0 versus 1–4	0.06	0.004	0.71	> 0.99	0.63	0.04
0–1 versus 2–4	0.02	0.0001	0.59	0.49	0.78	0.0007
0–2 versus 3–4	0.003	0.01	> 0.99	0.80	0.42	< 0.0001
0–3 versus 4	0.01	0.22	0.76	0.88	0.80	0.003
Number of parathyroid glands identified and preserved in situ						
0 versus 1–4	0.11	0.01	> 0.99	> 0.99	0.94	0.18
0–1 versus 2–4	0.07	0.002	0.79	0.34	0.95	0.002
0–2 versus 3–4	0.01	0.13	0.80	0.52	0.20	0.0005
0–3 versus 4	0.13	> 0.99	0.74	0.61	0.44	0.12

*HypoCa*, hypocalcaemia;

*HypoPTH*, hypoparathyroidism;

*PTH*, parathyroid hormone

When analyzed according to the number of parathyroids identified and preserved in situ, there was a trend for lower incidence of biochemical hypocalcaemia among patients with fewer parathyroids identified and preserved in situ, however, for most of the comparisons, the difference between groups was not significant, apart from that between patients with 0–2 versus 3–4 parathyroids identified (*p* = 0.01). In contrast, there was a significantly lower incidence of symptomatic hypocalcaemia among patients with 0 versus 1–4 (*p* = 0.01) and with 0–1 versus 2–4 parathyroids identified and preserved in situ (*p* = 0.002), but not between 0–2 versus 3–4 parathyroids identified, nor between 0–3 versus 4 parathyroids identified. There were no differences in permanent hypoparathyroidism between groupings.

Four hundred fifty-four patients had postoperative parathyroid hormone (PTH) levels available, of whom 258 also had preoperative PTH levels. There were no differences between groupings in preoperative PTH levels (Table 2). In contrast, a greater total number of parathyroids identified were associated with lower postoperative PTH levels. This difference was significant for all groupings (Tables 1 and 2). When considering numbers of parathyroids identified and preserved in situ, significant differences were seen between patients with 0–1 versus 2–4 parathyroids identified (*p* = 0.002), and with 0–2 versus 3–4 parathyroids identified (*p* = 0.0005) (Table 3).

Table 4 shows univariate analysis of all risk factors on calcaemia outcomes. Factors which were significantly associated with biochemical hypocalcaemia on univariate analysis were Graves disease, intraoperative identification of 2–4 parathyroids, intraoperative identification of 3–4 parathyroids, and intraoperative identification plus preservation in situ of 3–4 parathyroids. For symptomatic hypocalcaemia, Graves disease, age ≤ 65 years, incidental parathyroidectomy, intraoperative identification of 2–4 parathyroids, intraoperative identification plus preservation in situ of 2–4 parathyroids, and intraoperative identification of 3–4 parathyroids were significant. None of the study variables were significant for permanent hypoparathyroidism.

**Table 4:** Odds ratio and 95% confidence intervals for univariate risk factors for biochemical hypocalcaemia, symptomatic hypocalcaemia, and permanent hypoparathyroidism

	Biochemical hypocalcaemia	Symptomatic hypocalcaemia	Permanent hypoparathyroidism
Male sex	0.79 (0.44, 1.43)	0.68 (0.30, 1.56)	0.20 (0.01, 3.42)
Age > 65 years	0.77 (0.48, 1.24)	0.41 (0.19, 0.89)	0.54 (0.12, 2.40)
Graves disease	1.70 (1.12, 2.57)	2.18 (1.31, 3.64)	1.32 (0.45, 3.86)
Retrosternal	0.96 (0.60, 1.52)	0.81 (0.44, 1.52)	1.22 (0.39, 3.87)
Malignancy	1.54 (0.99, 2.41)	1.03 (0.56, 1.87)	2.34 (0.83, 6.59)
Specimen weight > 100 g	1.12 (0.70, 1.78)	0.92 (0.49, 1.72)	1.37 (0.43, 4.33)
Parathyroid in specimen	1.57 (0.93, 2.64)	1.88 (1.01, 3.49)	1.45 (0.40, 5.22)
2–4 parathyroids identified	1.61 (1.06, 2.46)	3.47 (1.74, 6.77)	0.77 (0.27, 2.15)
2–4 parathyroids identified and left in situ	1.46 (0.97, 2.20)	2.58 (1.40, 4.75)	0.87 (0.31, 2.44)
3–4 parathyroids identified	1.78 (1.22, 2.61)	1.98 (1.20, 3.26)	0.98 (0.36, 2.67)
3–4 parathyroids identified and left in situ	1.65 (1.13, 2.43)	1.52 (0.93, 2.49)	1.15 (0.42, 3.13)

On multivariate analysis, malignancy (OR 1.7, 95% CI 1.1, 2.8), Graves disease (OR 1.8, 95% CI 1.1, 2.7), and identification of 3–4 parathyroids (OR 1.7, 95% CI 1.0, 2.9) remained independently predictive of biochemical hypocalcaemia. For symptomatic hypocalcaemia, identification of 2–4 parathyroids (OR 5.9. 95% CI 1.5, 23.1), and identification of 3–4 parathyroids (OR 3.0, 95% CI 1.0, 9.1) were significant. Standardized coefficients of variables entered into the multivariate analysis are presented graphically in Supplementary materials.

## Discussion

In the present study, we found that identification of a greater number of parathyroid glands during TT did not result in any improvement in functional parathyroid preservation. In contrast, there appeared to be an increased incidence of both biochemical and symptomatic hypocalcaemia in cases with more parathyroids identified. Furthermore, identification of a greater number of parathyroids was found to be an independent predictor of both biochemical hypocalcaemia and symptomatic hypocalcaemia on multivariate analysis when other factors were controlled for.

The higher incidence of biochemical hypocalcaemia and symptomatic hypocalcaemia in cases with more parathyroid glands identified might raise the question as to whether dissection of parathyroid glands during identification is itself a risk factor for postoperative dysfunction, and might therefore advise against routine parathyroid identification. However, our findings were likely contingent on our surgical strategy of not routinely seeking parathyroid glands. It is possible that this meant that we were more likely to recognize glands with an inherently greater risk of intraoperative trauma or devascularisation, for example, glands located in exposed positions on the thyroid capsule; and less likely to recognize glands which were padded in fatty tissue adjacent to or away from the thyroid gland, which may be inherently less susceptible to trauma or devascularisation. Therefore, our findings may not be generalizable to series where the surgical strategy is to systematically identify as many parathyroids as possible. It is worth noting also that when biochemical hypocalcaemia was analyzed according to number of parathyroid glands identified and preserved in situ, taking into consideration glands which were autotransplanted, inadvertently removed, or visibly compromised, most of the differences in biochemical hypocalcaemia according to parathyroid identification were no longer significant. Therefore, we cannot conclude from our data that parathyroid identification per se is a risk factor for postoperative dysfunction.

Previous literature regarding the optimum strategy for parathyroid management during TT is controversial. Many authors have emphasized the need to routinely identify all 4 glands wherever possible [5, 9, 12–15]. However, others, similar to us, have reported that number of parathyroids identified did not correlate with calcaemia outcomes [4], or showed an inverse relationship [3, 10, 16]. An important drawback of many previously published studies is their retrospective nature. Such studies are weakened by reliance on retrospective documentation of parathyroid identification, which itself is very subjective. Among single-center prospective studies, Mehta et al., found no correlation between number of parathyroids identified and postoperative hypocalcaemia among a cohort of 265 cases, which also included 2-stage operations [4], while a previous report by our group found a higher incidence of hypocalcemia among patients with 3–4 parathyroid glands identified than those with 0–2 parathyroids identified among a cohort of 126 patients [17]. In contrast, Sitges-Serra reported a higher incidence of temporary and permanent hypoparathyroidism among cases with fewer parathyroid glands left in situ, although the incidences were not significantly different according to number of glands identified [9]. Similar findings were reported in a subsequent paper by the same group [15]. Some multicentre studies, using prospective observational design, have been reported, also with conflicting results. Those by the German Society of Surgery [5] and Scandinavian Quality Register [7] showed increased risk of hypocalcaemia with lower number of parathyroids identified, whereas that by the Italian Endocrine Surgery Units Association showed decreased incidence of temporary hypocalcemia in cases where parathyroids were not identified [6]. Drawbacks of such multicentre studies include cohorts comprising a mix of cases undergoing TT and subtotal thyroidectomy, operations performed by many different surgeons with variable techniques, and presumably considerable internal variability in definition of identified parathyroid glands, as well as incomplete follow up data.

Recently, optical techniques to aid parathyroid visualization using indocyanine green fluorescence have been reported [18, 19]. Such techniques may help reduce the incidence of postoperative hypocalcaemia compared to white light alone in cases where parathyroid glands are systematically sought [20]. It is possible going forward that evolution and application of these techniques may afford benefit to a strategy of systematic parathyroid identification. However, as yet there is no comparative data between TT with parathyroid identification using optical techniques and TT using capsular dissection technique without systematic parathyroid identification.

There are some limitations to our study which may limit its generalizability. Firstly, intraoperative determination of what constitutes parathyroid is highly subjective, and likely subject to significant interobserver variation, which can limit comparisons between different surgical series. Secondly, there is significant variability between thyroid surgeons in terms of surgical technique, and strategy for parathyroid management (i.e. systematically seeking or not seeking to identify all 4 glands in every case). Our findings may thus not be applicable to cases performed according to different techniques, to subtotal thyroidectomy cases, or cases where parathyroids glands are systematically sought intraoperatively. A third limitation is the possibility of evolution of surgical technique and criteria for parathyroid identification over time, within the series. In order to assess this possibility, we compared the average number of parathyroids identified per case in each 100 consecutive cases. These figures for 500 cases were remarkably similar (2.27, 2.28, 2.23, 2.24, and 2.17, respectively). Based on this, we feel that we were likely quite consistent throughout this series with regard to parathyroid identification. Fourthly, even though we prospectively recorded number of parathyroids identified, removed, devascularized, and reimplanted in every case, we did not consistently record the position of parathyroid glands, and thus we cannot provide evidence to support our theory that parathyroid glands located in certain exposed positions may be at inherently greater risk of postoperative hypofunction. A further limitation is that we did not include vitamin D status or renal function in our analysis, which could conceivably bias our findings. Finally, we are not able to compare our outcomes with a control group of cases undergoing systematic parathyroid identification, and therefore we cannot draw any conclusions regarding the superiority of one strategy over another.

On the other hand, one of the major advantages of our series was the prospective data collection, which facilitated accurate and consistent documentation of number of parathyroids in every case. The prospective data collection also facilitated inclusion of symptomatic hypocalcaemia as an outcome measure, which was not reported in many other series, and is perhaps a more clinically meaningful early outcome measure than biochemical hypocalcaemia. Other advantages were the likely internal consistency with regard to technique and definition of parathyroid identification; and the fact that patients were not routinely prescribed calcium in the perioperative period, which allowed us to assess postoperative hypoparathyroidism according to the gold standard of calcium levels and symptoms unhindered by exogenous calcium supplementation; and all patients having had calcium levels recorded for at least 2 postoperatively days, which maximized our capture of hypocalcaemic patients.

## Conclusion

In the present study, we found that intraoperative identification of a greater number of parathyroid glands did not lead to improvements in functional parathyroid preservation after TT. These findings would suggest that a strategy based on “watching out” for parathyroid glands without efforts to identify glands that are not immediately apparent is non-inferior to one based on systematic identification of all 4 parathyroid glands in every case. Variations in surgical cohorts, surgical techniques, definitions and management of hypocalcaemia, and subjectivity in parathyroid identification, may limit the generalizability of our findings.

## Supplementary Information

Below is the link to the electronic supplementary material.

Supplementary file1 (DOCX 17 KB)

## Data Availability

Anonymized data may be made available on reasonable request to corresponding author.
