# Supplementary material for: Association between number of parathyroid glands identified during total thyroidectomy and functional parathyroid preservation
Source: Langenbecks Arch Surg. 2021 Aug 18;407(1):297–303. doi: 10.1007/s00423-021-02287-6 (PMC8847165; doi:10.1007/s00423-021-02287-6)
Supplement: Supplementary file 1 — Supplementary file1 (DOCX 17 KB) [file 423_2021_2287_MOESM1_ESM.docx]

Supplementary Figures 1: Standardized coefficients for multivariate analysis for biochemical hypocalcaemia

Supplementary Figure 2: Standardized coefficients for multivariate analysis for symptomatic hypocalcaemia
